# Silymarin inhibits Toll-like receptor 8 gene expression and apoptosis in Ramos cancer cell line

**Published:** 2020

**Authors:** Nasrin Ranjbar, Ramin Saravani, Zohreh Faezizadeh

**Affiliations:** 1 *Student Scientific Research Center, Department of Clinical Biochemistry, School of Medicine, Zahedan University of Medical Sciences, Zahedan, Iran *; 2 *Cellular and Molecular Research Center, Department of Clinical Biochemistry, School of Medicine, Zahedan University of Medical Sciences, Zahedan, Iran*; 3 *Department of Clinical Biochemistry, School of Medicine, Zahedan University of Medical Sciences, Zahedan, Iran*; 4 *Department of Laboratory Sciences, Borujerd Branch, Islamic Azad University, Borujerd, Iran*

**Keywords:** Toll-like receptor, TLR8, Gene expression, Silymarin, Apoptosis, Ramos cells

## Abstract

**Objective:**

Silymarin is a herbal extract containing flavonolignans, and it has inhibitory effects against the growth of different cancer cell lines by inducing apoptosis. Toll-like receptors are suggested as a novel and attractive target to treat cancer. The current study aimed at examining the mechanism of silymarin-induced apoptosis in Ramos cells and investigating its effects on *TLR8* expression.

**Materials and Methods:**

The half maximal inhibitory concentration (IC_50_) of silymarin in Ramos cells was determined via MTT viability test while the type of cell death was tested by annexin V/propidium iodide (PI) double staining method. The activity of caspase-3 and expression of *TLR8* were measured in a time-dependent manner (in IC_50_) by colorimetric assay and real-time polymerase chain reaction (RT-PCR), respectively.

**Results:**

The results of MTT showed that IC_50_ of silymarin in Ramos cells was 100 μg/ml after 48 hr treatment (p<0.01). Flow cytometry by annexin V/PI, showed that silymarin induced early/late apoptosis in this cell line (p<0.05 to p<0.01). In addition, the caspase-3 colorimetric method showed that caspase-3 increased in the Ramos cell line after treatment (p<0.01). This treatment led to a reduction in *TLR8* mRNA expression in a time-dependent manner (p<0.01).

**Conclusion:**

The results indicated a new mechanism in the anticancer activity of Toll-like receptor (TLR) signaling after silymarin treatment in Ramos cancer cell line. This plant could be used to develop anticancer agents inhibiting TLRs.

## Introduction

Compounds derived from certain plants induce apoptosis in different cancerous cell lines (Unitt and Hornigold, 2011 ▶). It was reported that herbal agents suppress toll-like receptors (TLRs) and may be potential targets of chemotherapeutic compounds. The fruits and seeds of the medicinal plant *Silybum marianum *L., are used to obtain a bioactive extract named silymarin, which mainly contains silibinin and partially silibinin stereoisomers (Ramasamy and Agarwal, 2008 ▶;Wen et al., 2008 ▶). Extensive studies indicated strong chemopreventive and anticancer effects of silymarin on many types of cancers (Wen et al., 2008 ▶; Tyagi et al., 2007 ▶). There are more than 20 types of lymphatic system cancers. Burkitt lymphoma, a germinal center B-cell-derived cancer, is an important human oncogene found more than three decades ago. This cancer, which is a form of non-Hodgkin's lymphoma, begins in immune cells called B-lymphocytes (Burkitt, 1958 ▶). The most tenable hypothesis consistent with all the observed facts is that tumor formation results from interactions between some virus or viruses and a reticuloendothelial system altered by chronic and heavy infection by malarial or other parasites (Burkitt, 1969 ▶). 

TLRs are transmembrane pattern recognition receptors capable of recognizing generic pathogen-associated molecular patterns (PAMPs) (Barton and Kagan, 2009 ▶). Their activation induces intracellular signaling pathways that result in the production of a variety of nuclear factor (NF)-kB-mediated cytokines (e.g. tumor necrosis factor) and type I interferon (Kawai and Akira, 2011 ▶), which prevent cell death by expressing anti-apoptotic proteins such as Bcl-2 and induce chronic inflammation by producing cyclooxygenase-2 (Grimm et al., 2010 ▶; Bowie and O'neill, 2000 ▶). Humans have ten known functional TLRs, of which TLR 3, 7, 8, and 9 are expressed on intracellular vesicular membranes and are commonly involved in recognizing nucleic acids; *TLR8* detects viral single-stranded RNA (Kawai and Akira, 2006 ▶). Recently, it was observed that TLRs are expressed in tumor cells (Sato et al., 2009 ▶). The role of TLRs expression in tumor cells and its relationship with tumor development are exciting subjects in tumor immunity.

*TLR8* is activated in different cancers including cervical, pancreatic, and lung cancer cells (Zhang et al., 2014 ▶; Cherfils-Vicini et al., 2010a ▶; Grimmig, et al., 2015 ▶). Cherfils-Vicin et al. reported that *TLR8 *was expressed in human lung cancer cells, and PolyU (*TLR8* ligand) could upregulate B-cells lymphoma-2 (Bcl-2) and promote the survival of lung cancer cells. They also proposed that Bcl-2 plays a crucial role in autophagy and apoptosis pathways (Cherfils-Vicini et al., 2010b ▶). Therefore, *TLR8* is considered a key marker of tumor genesis and is a highly selective candidate to develop anti-proliferative and anti-malignant agents. To date, there is no report on *TLR8 *gene expression and the molecular mechanism involved in the anti-proliferative effect of silymarin in Ramos (P_53_-mutant) cancer cell line. Also, there are data on mutations in the P_53_ that associated with malignancy condition such as proliferation, migration, and invasion 

The current study aimed at examining the effect of silymarin on *TLR8 *gene expression and induction of apoptosis in Ramos cancer cell line.

## Materials and Methods

**Chemical reagents**

All the materials were of analytical grade. RPMI 1640 medium, trypan blue and trypsin were purchased from INOCLON (G. Innovative Biotech Co Inoclon), Iran). Fetal bovine serum (FBS), penicillin/streptomycin and amphotericin B were purchased from Gibco (Rockville, MD, USA). Annexin V/PI (propidium iodide**)**, apoptosis detection kit was obtained from BioVision (San Francisco, CA, USA). 3-(4, 5-dimethylhiazol-2-yl)-2, 5-diphenyltetrazolium bromide (MTT) and dimethyl sulfoxide (DMSO) were purchased from Sigma-Aldrich (St. Louis, MO, USA). Reverse transcription kit and real-time PCR kit were purchased from TaKaRa Bio Inc. (Dalian, China).

**Cell culture**

The P_53_-mutant Ramos cells (ATCC® Number: CRL-1596™; Organism: Homo sapiens, human; Cell Type: B lymphocyte; and Disease: Burkitt's lymphoma) were maintained in a medium that contained RPMI 1640 medium, fetal bovine serum (10%), penicillin (100 U/ml), and streptomycin (100 µg/ml). The P_53_-mutant Ramos cell line was grown under cell culture conditions at 37°C, with 95% humidity, with an atmosphere of 5% CO_2_.

**MTT viability assay**

To evaluate the cytotoxic effects of silymarin on Ramos cell line, cells were plated at a density of 6×10^3^ cell/well in a 96-well plate and exposed to various concentrations of silymarin (0, 25, 50, 75 and 100 μg/ml) for 24, 48 and 72 hr. Then, the viability of the cells was measured by MTT assay. Briefly, after the aforementioned periods, 20 μl of MTT dye was added and the solution was incubated at 37°C for 3 hr. Then, 200 μl of dimethyl sulfoxide (DMSO) was added and the solution was placed in a dark box for 15 min. Finally, all the plates were read at 570 nm wavelength using a microplate reader (Stat Fax 2100; Awareness Technology, Los Angeles, CA, USA).

**Flow cytometry assay using annexin V/PI staining**

Apoptosis was detected using annexin/PI staining kit. Ramos cells were seeded (1 × 10^5^ cell/well) in 6-well plates and incubated for 12 hr before treatment with increasing concentrations of silymarin. After 48 hr of treatment, both treated and untreated cells were trypsinized, washed with cold phosphate-buffered saline (PBS), and resuspended in PBS. Apoptosis was measured using an annexin V/PI double staining kit (BioVision, San Francisco, CA, USA). According to the manufacturer’s instructions, first of all, the cell pellets were stained with 250 μl 1X binding buffer and later, incubated with 2.5 µl annexin V for 15 min in the dark at room temperature. Before analysis, 2.5 µl of propidium iodide (PI) was added and incubated for 10 min. Finally, the working solution (1000 µl) was added to the samples before analysis by a fluorescence-activated cell sorting (FACS) cytometer (BD Biosciences, San Jose, CA, USA).

**Caspase-3 activity**

Caspase-3 activity was measured according to the manufacturer’s instructions using colorimetric Assay Kits (R&D systems Co. Germany). Briefly, cells were treated with silymarin (IC50) at different times (0 [control], 6, 12 and 24 hr); next, cells were washed twice in cold PBS and lysed using lysis buffer for 10 min. Then, cells were centrifuged at 10000 g for 10 min and then 50 µl reaction buffer and 5 µl of caspase-3 substrate were added to supernatants and incubated at 37°C for 1 hr. The results of absorbance as measured by a microplate reader (Stat Fax 2100; Awareness Technology, Los Angeles, CA, USA) at 405 nm wavelength are expressed as fold changes of caspase-3 activity in treated cells divided by the caspase-3 activity of adjacent untreated cells.

***TLR8*** **mRNA expression**

Cells at a density of 12×10^4^ cells/well were seeded in 96-well plates and then they were incubated without or with silymarin (IC50) at different times (0 [control], 4, 8, 12, 24, and 48 hr) and RNAs were extracted by RNX (SinaClon, Tehran, Iran). After synthesis of cDNAs by reverse transcription kit (TAKARA Bio Inc. Dalian, China) based on the extracted RNAs, the mRNA expression levels of *TLR8 *gene were evaluated by real-time PCR (Applied Biosystems, Foster City, CA, USA) using specific primers for both target genes [*TLR8 *mRNA] and *GAPDH* used as housekeeping gene (Table 1).

**Statistical analysis**

 All data were analyzed by IBM SPSS statistics version 22 (SPSS Inc., Chicago, IL, USA). To compare data, One-way analysis of variance (ANOVA) with the Dunnett’s test was used. A p-value of less than 0.05 was considered statistically different. 

**Table 1:** Sequences of the primer pairs for *TLR8 *and reference gene

Target name	Amplicon length	Primer sequence forward Primer sequence reverse
*TLR8*	184 bp	5ʹCAGAGCATCAACCAAAGCAA3ʹ 5ʹGCTGCCGTAGCCTGAAATA3ʹ
*GAPDH*	150 bp	5ʹGAGCCACATCGCTCAGACAC3ʹ 5ʹCATGTAGTTGAGGTCAATGAAGG3ʹ

## Results

**Anti-proliferative effect of** **silymarin** **on human Ramos cancer cells**

**Figure 1:**
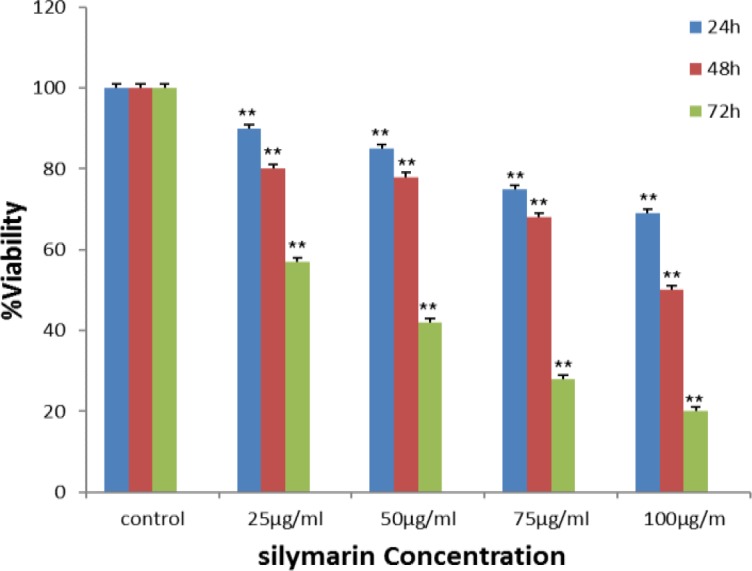
Effects of silymarin on suppression of Ramos cell growth. Cells were exposed for 24, 48 and 72 hr to different concentrations of silymarin, and cell proliferation was examined by MTT assay. This compound decreased cell proliferation in time- and dose-dependent fashions. **p<0.01 show significant differences compared to control cells

The effect of silymarin on the Ramos cell line was examined. The cells in different concentrations of silymarin and at different times were exposed, the cell line viability was examined by using an MTT assay. The most significant inhibitory effects of silymarin in Ramos cancer cell line were 69±4.5%, 50±2.1% and 19±1.5 at concentration 100 µg/ml after treatment for 24, 48 and 72 hr, respectively. Concentration of 100 μg/ml at time 48hr revealed decreased growth by 50% in Ramos cancer cell line (p<0.01). Figure 1 shows the results of MTT in cancer cell line.

**Detection of apoptosis by flow cytometry**

**Figure 2:**
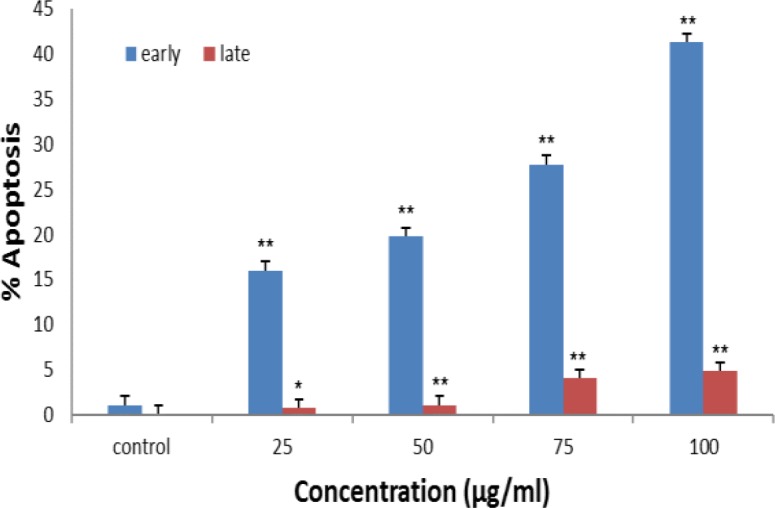
Flow cytometric evaluation of apoptosis in Ramos cells after 48 hr treatment with silymarin by staining with annexin-V and propidium iodide (PI). Both early and late apoptosis were increased in cells in comparison with untreated cell line. *p<0.05 and **p<0.01 show significant differences compared to control cells

**Figure 3:**
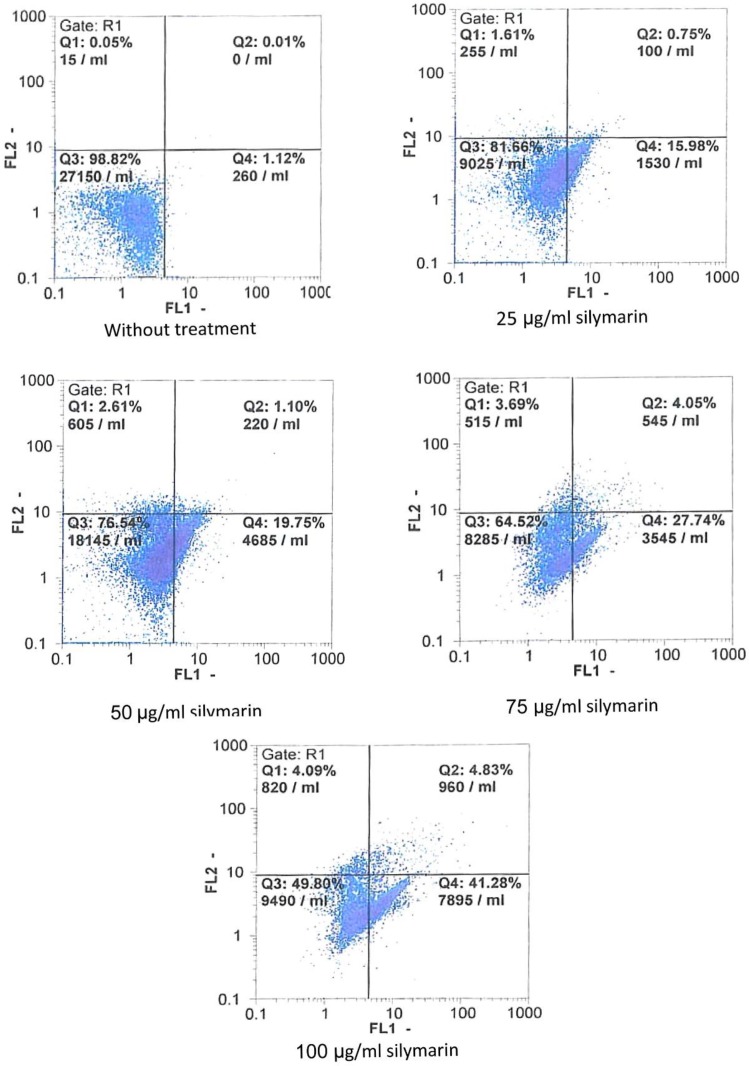
Silymarin induced apoptosis in Ramos cell line. Cells were treated with different concentrations of silymarin (0 [control], 25, 50, and 100 µg/ml). After 48 hr of treatment, Ramos cells were stained with annexin/PI. All measurements done by flow-cytometry were performed independently in triplicates per experimental point, and representative results are shown

To study whether silymarin induced apoptosis in the cell line, both treated and untreated Ramos cells were double-stained with annexin V-FITC/PI; then, cells were analyses by flow cytometry. The obtained results showed a significant increase in the percentage of both early and late apoptosis in a concentration-dependent manner (p<0.05 to p<0.01); Figures 2 and 3).

**Caspase-3 activation assay**

**Figure 4:**
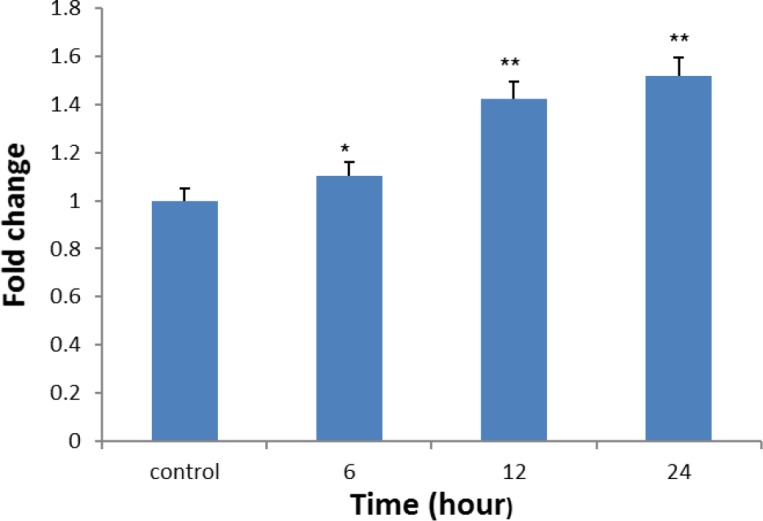
Colorimetric assay of caspase-3 activity after treatment with silymarin 100 μg/ml. Activity of caspase-3 was enhanced in Ramos cancer cells. *p< 0.05 and **p<0.01 show significant differences compared to control cells

 To examine the role of caspase-3 in silymarin*-*induced apoptosis, the activity of caspase-3 was measured. Treatment of Ramos cancer cells with 100 µg/ml of silymarin markedly increased the activity of caspase-3 in a time-dependent manner (p<0.05 to p<0.01). Figure 4 shows Colorimetric assay of caspase-3 activity in cancer cell line.

**RT- PCR assay**

**Figure 5:**
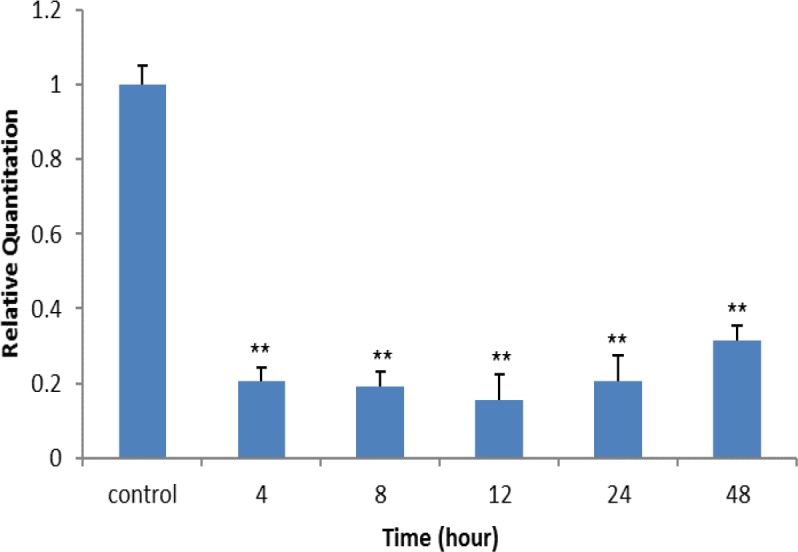
Real-time RT -PCR analysis of TLR8 mRNA expression on human Ramos cancer cell line following silymarin (100 μg/ml) treatment in different times. **p<0.01 show significant differences compared to control cells

The expression levels of TLR8 were examined by real-time RT-PCR using SYBR Green in Ramos cancer cells. As shown in Figure 5, TLR8 mRNAs in cancer cell line was detected, and also the effects of silymarin on expression of the TLR8 gene was performed at different times (o[control], 4, 8, 12, 24 and 48 hr). The results show that TLR8 gene expression level decreased significantly in Ramos cancer cell line in the presence of silymarin (100µg/ml) in comparison to untreated cells (p<0.01). 

## Discussion

The current study focused on the effect of silymarin on *TLR8 *expression inhibition, cell viability, and induction of apoptosis in Ramos cancer cell line. The results of the current study showed a significant positive correlation between Ramos cancer cell line with silymarin and reduced expression of *TLR8 *and apoptosis in a concentration- and time-dependent fashion; silymarin also increased caspase-3 activity.

Silymarin is composed of several flavonolignans including silybin A, silybin B, isosilybin, and silychristin. The main components of *Silybum marianum* such as polyphenolic fraction and silybin have significant antioxidant and cytoprotective effects (Křen and Walterova, 2005 ▶; Pradhan and Girish, 2006 ▶). Previous investigations showed that silymarin induces apoptosis in different cell lines such as HepG2 (Ramakrishnan et al., 2009 ▶), PC-3 (Deep et al., 2008 ▶), C33A, HeLa (Huang et al., 2005 ▶; Yu et al., 2012 ▶), and FaDu cell line, as well as oral cancer (Su et al., 2013 ▶). So far, extensive studies demonstrated that herbal compounds could inhibit inflammatory responses by the modulation of TLRs (Romano et al., 2013 ▶) and finally, induce apoptosis (Marx, 2004 ▶; Kelly et al., 2006 ▶). TLRs play a pivotal role to maintain tissue homeostasis by regulating tissue repair and inflammatory responses to injuries (Chuang and Ulevitch, 2000 ▶). 

Through activating the NF-kB pathway, TLRs play an essential role in inflammatory processes (Grimmig et al., 2015 ▶). Thus, targeting TLRs should be considered a potential strategy to treat cancers (Grimmig et al., 2015 ▶). *TLR8 *gene is mapped to chromosome Xp22 (Wang et al., 2008 ▶). Recently, overexpression of *TLR8* was reported in a variety of tumor cells (Pradere et al., 2014; Rivlin et al., 2011) such as pancreatic cancer (Grimm et al., 2010 ▶). Previous studies demonstrated that the expression levels of *TLR7*/*TLR8 *genes were associated with colorectal cancer (tumor progression) and that survival rate was lower among patients with *TLR7/TLR8 *overexpression (Grimm et al., 2010 ▶). Mutations in the *P*_53_ gene are observed in almost 10% of hematopoietic malignancies (Brosh and Rotter, 2009 ▶). Also, P_53_-mutant cells show different results on growth inhibition as compared with wild-type P_53 _(Farrell et al., 1991 ▶). On the other hand, studies revealed that binding to human papillomavirus 16 E6 oncoproteins leads to rapid proteolytic degradation of P_53_ and induces the ubiquitin pathway in cells. However, Ramos cells are resistant to E6-directed degradation and are Epstein-Barr virus-negative (EBV-negative). Also, these cells express high levels of TLR8 and could be used as a suitable model to investigate the functions and mechanisms of TLRs in *in vitro* studies (Vousden et al., 1993 ▶). However, mutant P_53_ downstream signaling pathway is not clear because there are different P_53_-mutant variants (Rivlin et al., 2011 ▶). TLRs ligand binding induces myeloid differentiation primary response 88 (MYD88) adapter molecules, which leads to the activation of mitogen-activated protein kinase (MAPK) signaling pathway, and in turn, inhibition of apoptosis via expression of anti-apoptotic proteins such as Bcl-2 (Grimm et al., 2010 ▶; Bowie and O'neill, 2000 ▶). In the current study, it was observed that different concentrations (25–100 µg/ml) of silymarin induce apoptosis in Ramos cell line (P_53_-mutant).A study revealed that silymarin induces apoptosis and causes drug sensitivity in anaplastic lymphoma cells (Molavi et al., 2016 ▶). It is also reported that silymarin induces apoptosis in silymarin-treated K562 cells (Faezizadeh et al., 2012 ▶).

Caspase-3 plays a vital role to induce apoptosis (Hussein, 2005 ▶). The activation of caspase-3 from its zymogen form (procaspase-3) is probably triggered through two mechanisms including the release of cytochrome c (intracellular way) and activation of caspase-8 (extracellular way) (Wang et al., 1999 ▶). Induction of apoptosis is considered an anti-cancer mechanism for many bio-active agents. *Epimedium* contains Icariin, the hydrolytic product of which exhibits induction of cell apoptosis by activation of caspases in human Raji Burkitt lymphoma and P3HR-1 cell line (Li et al., 2014 ▶). The application of plant compounds, particularly silymarin, as chemotherapeutic compounds for cancers is recommended since they do not impose any adverse effects on normal lymphocytes (Agarwal et al., 2006 ▶; Shanmugam et al., 2011 ▶). Therefore, targeting TLRs could be a promising strategy to expand anticancer agents. 

In summary, the current study data indicated downregulation of *TLR8 *expression after silymarin treatment in Ramos cancer cells. It was also discovered that silymarin effectively suppressed cell proliferation, induced apoptosis in Ramos cancer cells in a concentration-dependent fashion, and caused activation of caspase-3. Ultimately, according to the obtained results, TLR8 signaling may be a potential target for Burkitt lymphoma therapy.
